# Oxidative Stress and DNA Damage in Chronic Disease and Environmental Studies

**DOI:** 10.3390/ijms21186936

**Published:** 2020-09-21

**Authors:** Marco Peluso, Valentina Russo, Tommaso Mello, Andrea Galli

**Affiliations:** 1 Research Branch, Regional Cancer Prevention Laboratory, ISPRO-Study, Prevention and Oncology Network Institute, 50139 Florence, Italy; valrusso.bio@gmail.com; 2 Department of Experimental and Clinical Biomedical Sciences, University of Florence, 50139 Florence, Italy; tommaso.mello@unifi.it (T.M.); a.galli@dfc.unifi.it (A.G.)

Humans are continually exposed to a large number of environmental carcinogens [1], some of them share a specific toxicity model which acts via the enhancement of cellular levels of reactive oxygen species (ROS), such as superoxide anion and hydroxyl radicals [2]. ROS are chemicals capable of inducing oxidative stress, a condition where free radical generation overwhelms antioxidant protection in the body’s cells, causing oxidative damage of proteins, lipids and nucleic acids [2]. One biological consequence is the decline in physiological mechanisms designed to maintain cell repair and metabolic homeostasis, which can lead to tissue injury and cell transformation [3]. Oversensitive reactions to endogenous and extrinsic agents are associated with phenotypes characterized by elevated levels of genetic alterations [4,5], a process that can initiate carcinogenesis [6]. From a general perspective, the study of these factors can be useful to develop personalized medicine and to fill the knowledge gap of the molecular mechanisms behind the pathogenesis of chronic diseases. In this context, the current Special Issue’s challenge is to provide an overview on the topic of ROS, oxidative DNA damage and related-DNA repair factors in chronic diseases and environmental studies. 

The study by Vodicka et al. [7] analyzed the role of DNA damage and DNA repair mechanisms in colorectal carcinogenesis. In colorectal cancer (CRC), ROS overproduction can induce oxidative stress and oxidative DNA damage, that, if unrepaired, can cause mutations, including G>T transversion, leading, ultimately, to cancer. Evidence of the relationship between oxidative stress and CRC is coming from *MUTYH*-associated polyposis and *NTHL1*-associated tumor syndrome. The latter are two hereditary syndromes whose germline mutations cause the loss-of-function in glycosylases, a DNA repair defect which induces oxidative DNA damage accumulation and the transition from early adenoma to malignant cells. Other support for the oxidative stress hypothesis is provided from genetic studies showing an association between polymorphisms in base excision repair (BER) genes, e.g., 326Ser/Cys *OGG1*, 324Gln/His and 324His/His *MUTYH* genotypes and CRC risk as well as a link between the rates of BER-individual DNA excision repair capacity in non-malignant adjacent mucosa and the rates of overall and relapse-free survival in CRC. Furthermore, a diet rich in antioxidants and bioactive compounds appears to modify CRC risk, e.g., by increasing repair capacity, by decreasing DNA damage or by modifying the metabolic profile of gut microbiome. Specifically, the human microbiome can affect CRC risk through the production of ROS and genotoxic compounds or through the activation of pro-inflammatory cascades and cellular transformation. Thus, CRC treatment could be improved in various ways, e.g., by targeting ROS production and antioxidant defense or by exploiting acquired/inherited defects in DNA repair together with the use of conventional chemotherapeutics. 

The review by Bhardwaj et al. [8] addressed the study of abnormal energy metabolism in cancer cells that show a dependence on glycolysis, a cytoplasmic pathway that generates ATP, rather than on mitochondrial oxidative phosphorylation in the presence of oxygen for their energy requirement. In tumors, energy metabolic alterations are associated with the high production of ROS levels, which need to be kept under certain limits by increasing the action of the glycolysis pathway, the pentose phosphate pathway and the tricarboxylic acid cycle in order to maintain redox homeostasis and to prevent ROS-mediated cancer cell death. In tumors, the interplay between alterations of energy metabolism and ROS production plays a major role in regulating the tumor’s response to chemotherapeutic drugs. In particular, tumor resistance can be induced by various mechanisms, e.g., by high activity of p-glycoprotein, an important ATP-binding cassette transporter involved in pumping chemotherapeutic drugs out of cells, by hexokinase 2 overexpression which confers drug resistance enhancing ROS-induced autophagy, by stimulating the pentose phosphate pathway activity or by inducing high levels of glucose 6-phosphate dehydrogenase. Therefore, targeting metabolic deregulations in tumors with personalized cancer treatments could enhance the tumor’s response to chemotherapeutic drugs by using inhibitors capable of blocking the MEK/ERK pathway enhanced by the hexokinase 2 activity or by the inhibition of glucose 6-phosphate dehydrogenase with chemical inhibitors. 

In the review by Lee et al. [9], large interest was on the study of the repair of oxidative DNA damage via the nucleotide excision repair (NER), rather than through the classical BER pathways. In simple terms, ROS-induced oxidative DNA damage can be grouped into two categories (a) non-bulky single-base lesions, if the damage is associated to non-helix distorting modifications, and (b) bulky lesions, if the DNA damage causes helix distorting lesions. On one hand, the BER pathway is charged to repair non-bulky single-base lesions in the form of small chemical adducts, such as oxidation, alkylation and deamination damage; on the other hand, NER fixes bulky oxidative lesions, such as purine 5’,8-cyclonucleosides, interstrand cross-links and DNA–protein cross-links. NER factors can also participate in the BER process of lesion recognition and interact with BER proteins to stimulate enzyme activity and improve DNA repair rates. Hence, newly therapeutic treatments could be developed by targeting DNA repair defects or by using small molecule inhibitors or modulators of NER/BER factors. 

To investigate whether the accumulation of ROS-induced DNA damage and defective DNA damage response (DDR) rates are associated with chronic myeloid leukemia (CML), a myeloproliferative neoplasm, the study by Popp et al. [10] examined the levels of double strand breaks (DSBs) and DDR in CML patients by immunofluorescence microscopy and Western blotting. High DSB production, detected by the γH2AX foci analysis, was found in chronic, accelerated and blast phases of CML patients as well as in those with loss of major molecular response in respect to controls or CML patients with deep molecular response. Increased frequencies of erroneous non-homologous end joining and microhomology-mediated end joining repair mechanisms, measured by the co-localization of γH2AX/53BP1 foci, were observed across the spectrum from the chronic towards the blast phases of CML. DDR decline was also detected by the defective expression of (p-)ATM and (p-)CHK2. Lastly, the development of genetic instability in the CML appears to be due to DNA damage accumulation in the course from the chronic phase of CML towards the blast phase of CML and, more importantly, DNA damage together with the progressive activation of error-prone DSB repair mechanisms and the DDR decline could play a role in the blastic transformation and the disease progression of CML. 

The study by Souliotis [11] addressed the analysis of the interplay between DNA damage response and repair (DDR/R) with the innate immune response to endogenous and exogenous agents, in order to understand how DDR/R deregulation can act together with immune activation in the pathogenesis of systemic autoimmunity. In this field, evidence of the interaction between DNA metabolism and innate immune response was initially provided from the Aicardi–Goutières syndrome, a disease characterized by mutations in RNaseH2, a protein that removes ribonucleotides from DNA to maintain DNA integrity. Further evidence was obtained from the telengiectasia, an autosomal recessive disorder characterized by mutations in the *ATM* gene, a defect that can cause chronic accumulation of DNA damage capable to activate the immune system. The occurrence of an interplay between DDR/R defects and the innate immune response was also demonstrated in systemic lupus erythematosus, an autoimmune disease where patients carry mutations in DNA repair enzymes, DNA damage accumulation, high rates of apoptosis and increased levels of antibodies against DNA repair proteins. Moreover, this interplay was also found in the pathogenesis of rheumatoid arthritis. Therefore, new therapies against autoimmune diseases could be developed targeting this interaction, e.g., by utilizing DDR/R inhibitors, such as HDACi, givinostat, or vorinostat, or by applying a combination of p53 activators and CHK1/2 inhibitors. 

In the effort to explain the higher incidence of thyroid cancer in populations who live near volcanos, the review by Malandrino et al. [12] examined the toxic effects caused by exposure to heavy metals, chemical compounds capable of inducing ROS overproduction, in volcanic areas. Interestingly, the concentrations of several heavy metals were not increased in water and lichen, whereas elevated amounts of boron, tungsten, molybdenum and palladium were detected in the subjects who were living in residential areas near the Etna volcano, in Sicily, Italy. To explain how small increases in environmental metals can be associated to an enhanced risk of thyroid cancer in volcanic areas, authors suggested that this relationship was more mediated by hormesis effects resulting in inverted U-shaped dose-response curves rather than a linear dose-response relationship. Additionally, life-long and selective accumulation of one or more heavy metals could be behind the carcinogenic effects that are predominantly found in the thyroid gland. 

The study by Kucharova et al. [13] evaluated the genotoxic effects of anesthetic substances used for general anesthesia or neuraxial anesthesia treatments in a cohort of patients undergoing orthopedic traumatological surgery. The frequency of single-strand DNA breaks, oxidized pyrimidine bases and oxidized purine bases was measured by comet assay before and after anesthesia procedures. High levels of DNA damage were detected in patients undergoing general anesthesia, indicating that halogenated gases isoflurane and sevoflurane can induce oxidative DNA damage, whereas the treatment with epidural and subarachnoid anesthesia was not toxic.

Cellai et al. [14] examined the genotoxic mechanisms underlying the development of nasal and sinonasal cancer (SNC) in occupational settings characterized by carcinogenic exposure to wood dust, a recognized cause of SNC. In that study, the association between the generation of 3-(2-deoxy-α-d-erythro-pentafuranosyl)pyrimido[1,2-β]purin-10(3H)-one deoxyguanosine, a major-peroxidation-derived DNA adduct, and the wood dusts was analyzed in woodworkers in respect to controls living in Tuscany, Italy, by chromatographic and mass spectrometry techniques. High levels of oxidative DNA damage were found in woodworkers exposed to average levels of 1.48 mg/m^3^ wood dust, especially when concomitant carcinogen co-exposure occurred. 

In the exciting exploration of the anti-genotoxic effects of dietary flavonoids, the study of Jee et al. [15] analyzed the beneficial properties of silymarin, a natural flavonoid known for its anti-oxidant and anti-inflammatory properties, in relation to the exposure to airborne carcinogens. Specifically, the toxicity of benzo[a]pyrene (B[a]P), a carcinogen contained in tobacco smoke and air pollution and capable of causing both DNA damage and ROS production, was analyzed by ELISA, HPLC, immunofluorescence and Western blot in experimental cells. The co-treatment of B[a]P with silymarin significantly reduced the levels of DNA damage, indicating that this natural flavonoid has antigenotoxic properties. Interestingly, the protective property of silymarin was mainly related to its capacity of up-regulating the expression of *Nrf2* and *PxR* rather than to antioxidant effects. 

In conclusion, the collection of this Special Issue covers topics representative of several key research areas in the field of cancer, autoimmune diseases and environmental studies. Further effort is indeed needed for developing innovative models of therapy—based on personalized medicine—that identify the most efficient therapeutic intervention for patients with low response to chemotherapeutics. For example, chemotherapeutic response could be improved by targeting the overproduction of ROS in malignant cells to reduce multidrug resistance or by using modulators of NER/BER proteins acting against acquired or inherited defects in DNA repair pathways. Therapies for autoimmune diseases could also benefit from utilizing DNA repair inhibitors and/or p53 activators. Next, the collection shows that DNA damage accumulation and/or DNA repair can be behind the blastic transformation and the disease progression of CML; that general anesthesia as well as the environmental exposure to wood dust can induce DNA damage; and that dietary flavonoids can act against the production of DNA damage. Lastly, inverted U-shaped dose-response curves and/or life-long and selective accumulation of heavy metals could be associated with the risk of thyroid cancer in populations living in volcanic areas. The data produced in those and future studies could be translated in the real environment to develop new therapies, to fill knowledge gaps and to be the source for further methodological and application advances. 
